# Efficiency of Promoters of Human Genes *FAP* and *CTGF* at Organism Level in a *Danio rerio* Model

**DOI:** 10.3390/ijms24087192

**Published:** 2023-04-13

**Authors:** Polina I. Selina, Irina V. Alekseenko, Anastasia I. Kurtova, Victor V. Pleshkan, Elena E. Voronezhskaya, Ilya V. Demidyuk, Sergey V. Kostrov

**Affiliations:** 1 National Research Center “Kurchatov Institute”, 123182 Moscow, Russia; 2 Shemyakin-Ovchinnikov Institute of Bioorganic Chemistry RAS, 117997 Moscow, Russia; 3 Koltsov Institute of Developmental Biology RAS, 119334 Moscow, Russia

**Keywords:** connective tissue growth factor, drug development, fibroblast activation protein alpha, genetic therapy, zebrafish

## Abstract

The identification of tissue-specific promoters for gene therapeutic constructs is one of the aims of complex tumor therapy. The genes encoding the fibroblast activation protein (*FAP*) and the connective tissue growth factor (*CTGF*) can function in tumor-associated stromal cells but are practically inactive in normal adult cells. Accordingly, the promoters of these genes can be used to develop vectors targeted to the tumor microenvironment. However, the efficiency of these promoters within genetic constructs remains underexplored, particularly, at the organism level. Here, we used the model of *Danio rerio* embryos to study the efficiency of transient expression of marker genes under the control of promoters of the *FAP*, *CTGF*, and immediate early genes of *Human cytomegalovirus* (CMV). Within 96 h after the injection of vectors, the *CTGF* and CMV promoters provided similar equal efficiency of reporter protein accumulation. In the case of the *FAP* promoter, a high level of reporter protein accumulation was observed only in certain zebrafish individuals that were considered developmentally abnormal. Disturbed embryogenesis was the factor of changes in the exogenous *FAP* promoter function. The data obtained make a significant contribution to understanding the function of the human *CTGF* and *FAP* promoters within vectors to assess their potential in gene therapy.

## 1. Introduction

To date, sufficient data confirm the significant role of many cell types of different origins associated with cancer in the growth of malignant neoplasms [1]. Such support for cancers impairs the efficacy of treatment targeted at proper cancer cells in practical medicine. The complex therapeutic approach targeted at several elements of a cancer lesion is topical in oncotherapy. Accordingly, the identification of genes activated in the stroma of various tumors relative to normal tissues and correlating with tumor growth and expansion are among the key factors for drug development. The regulatory elements of such genes can be used to construct expression vectors. In this context, genes of the connective tissue growth factor (CTGF, extracellular matrix protein) [2,3,4,5] and the fibroblast activation protein (FAP, transmembrane peptidase) [6,7,8,9] can be promising sources of regulatory sequences.

It is necessary to notice that the activity of these genes is manifested under conditions that require changes in the structure of the extracellular matrix (ECM) and stroma transformation. Similar to other type-II transmembrane serine proteases, FAP interacts with structural ECM proteins and cell surface integrins [10,11], which modulates the adhesive and migration properties of cells. In addition, FAP indirectly stimulates cell proliferation through increased expression of p-ERK [11]. The *CTGF* gene product mediates a wide range of processes; in particular, it was shown to modulate the expression of the main component of structural matrix fibers of type-I collagen, angiopoietin-2 in angiogenesis as well as the gene of matrix metalloproteinase MMP-2, which is also involved in the ECM remodeling [12,13,14]. CTGF induces the proliferation of fibroblasts, stellate cells, and cells of epithelial origin [15,16,17,18]. CTGF interaction with the vascular endothelial growth factor interferes with its interaction with the corresponding receptor, which leads to the differentiation of mesenchymal stem cells into fibroblasts [19].

Thus, FAP and CTGF are among the key elements of homeostasis in stromal tissues. Accordingly, both the proteins and their genes can be therapeutic targets in the reactive stroma, e.g., fibrosis, rheumatoid arthritis, and many cancer types including breast, pancreas, and hepatocellular cancers [2,3,4,5,6,7,8,9,20,21,22,23,24,25,26,27]. At the same time, the regulatory sequences of the *FAP* and *CTGF* genes can be used for the targeted expression of drugs in stromal tissues. The *FAP* and *CTGF* genes are most active in activated fibroblasts including myofibroblasts and tumor-associated fibroblasts. However, their expression can be detected in cardiomyocytes, pericytes, and endothelial cells in regenerating tissues [25,28,29,30]; in this context, the constructs based on *FAP* and *CTGF* genetic elements can also be used in regenerative medicine.

The function of human *CTGF* and *FAP* promoters in vector systems remains underexplored. These regulatory elements were characterized in experiments with the primary and continuous cultures. They provided for low but tissue-specific expression relative to high constitutive promoters [31,32]. To date, their function at the organism level is not sufficiently described.

It should be noted that vector entry into different cell populations in conditions of continuous variation of the cell expression profile and interaction between cell populations can modulate the therapeutic efficiency of the constructs used in medical practice. Taking this into account, experiments on organism-level models are an essential stage in the development of biomedical genetic constructs. In particular, the efficiency of target protein synthesis under the control of the *CTGF* and *FAP* regulatory elements in a system with multiple cell populations can significantly contribute to the evaluation of their clinical potential.

Zebrafish (*Danio rerio*) is one of the successful organism models in various biomedical studies since it shares many processes with all vertebrates. High fecundity and rapid development relative to other organism models make zebrafish a convenient organism-level screening system. As demonstrated previously, the zebrafish model makes it possible to evaluate the basic properties of vector systems such as time-related accumulation and tissue distribution of the target protein as well as tissue-specificity and toxicity of the vector and target expression product [33,34,35], which substantiated the use of this model system to evaluate the expression efficiency of the promoter elements. Here, we used the model of developing zebrafish embryos to comparatively analyze the efficiency of human *FAP* and *CTGF* promoters as well as that of immediate early genes of *Human cytomegalovirus* (CMV) in nonviral vectors with *Photinus pyralis* luciferase gene and enhanced green fluorescent protein (EGFP) gene as reporter ones.

## 2. Results

### 2.1. Quantitative Analysis of Expression Vector Efficiencies at Organism Level

The efficiency of promoters at the organism level was quantified by the marker protein luciferase of the firefly *Photinus pyralis*. The luciferase gene expression was controlled by the *FAP*, *CTGF*, or CMV promoter. Since the synthesis of active luciferase requires no post-translational modification, the level of the protein synthesized in eukaryotic cells is directly proportional to its enzyme activity and the luminescence signal of the reaction with the natural substrate added to embryonic lysates. The reference vectors pGL3-basic vector (pGL3-bv) with no promoter sequence and pGL3(CMV-luc) with a strong constitutive promoter of immediate early genes of *Human cytomegalovirus* were used as controls.

**Figure 1:**
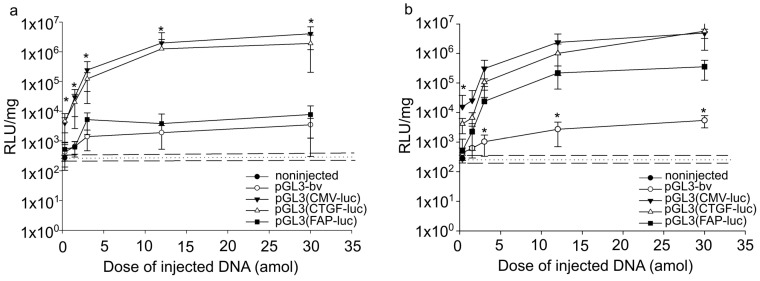
Luciferase activity in zebrafish embryos 24 h (**a**) and 96 h (**b**) after injection. Abscissa: quantities injected vectors pGL3(CMV-luc), pGL3(CTGF-luc), pGL3(FAP-luc), and pGL3-bv. Ordinate, relative luminescence units (RLU) normalized to 1 mg total protein in embryonic lysates, logarithmic scale. The dotted line indicates the average luminescence level in lysates of uninjected embryos, and the dashed lines indicate the size of error bars. (**a**) Significant differences in the luciferase activity after the injection of pGL3(CMV-luc) and pGL3(CTGF-luc) relative to vectors pGL3(FAP-luc) and pGL3-bv were observed over the whole DNA dose range (*p* < 0.05). (**b**) Significant differences in the luciferase activity after the injection of pGL3(FAP-luc), pGL3(CMV-luc), and pGL3(CTGF-luc) relative to vector pGL3-bv were observed from 3 to 30 attomoles DNA dose range (*p* < 0.05). The values were averaged for three independent experiments with three replicates for each point (*n* = 27). Error bars indicate standard deviation (SD), * *p* < 0.5.

The luciferase activity exceeding the background level (5 × 10^2^ relative light units per mg total protein) was detected 24 h after the injection of pGL3(CMV-luc), pGL3(CTGF-luc), pGL3(FAP-luc), or pGL3-bv in the embryos. The signal level depended on the plasmid dose (Figure 1a). For doses from 0.3 to 12 attomoles of vector DNA per embryo, luciferase activity increased for all plasmids and reached a plateau at higher doses.

pGL3(CMV-luc) and pGL3(CTGF-luc) provided for similar luciferase activities 24 h after injection. The luciferase activity after the injection of 0.3 attomoles of plasmids was 10 times that for the reference vector pGL3-bv. Further raising the DNA dose (from 1.5 to 30 attomoles) increased the difference from pGL3-bv to two orders of magnitude. In the case of pGL3(FAP-luc), the corresponding luciferase activity was comparable to that of the reference vector pGL3-bv.

In the period from 24 to 96 h after the injection, the luminescence signal in the embryos injected with 0.3–30 attomoles of pGL3(CMV-luc), pGL3(CTGF-luc), or pGL3-bv did not significantly differ. In the dose range from 0.3 to 1.5 attomoles, luciferase activities for pGL3(FAP-luc) and pGL3-bv were similar. However, higher doses (above 3 attomoles) increased the luciferase activities for pGL3(FAP-luc) 10-fold (Figure 1b).

Thus, *CTGF* and CMV promoters provided similar enhanced marker gene expression. The accumulation of marker proteins for pGL3(FAP-luc) increased in zebrafish embryogenesis, and by 96 h after injection approached the levels for the CMV and *CTGF* regulatory elements.

### 2.2. Quantitation of Embryonic Cells Expressing Marker Protein

**Figure 2:**
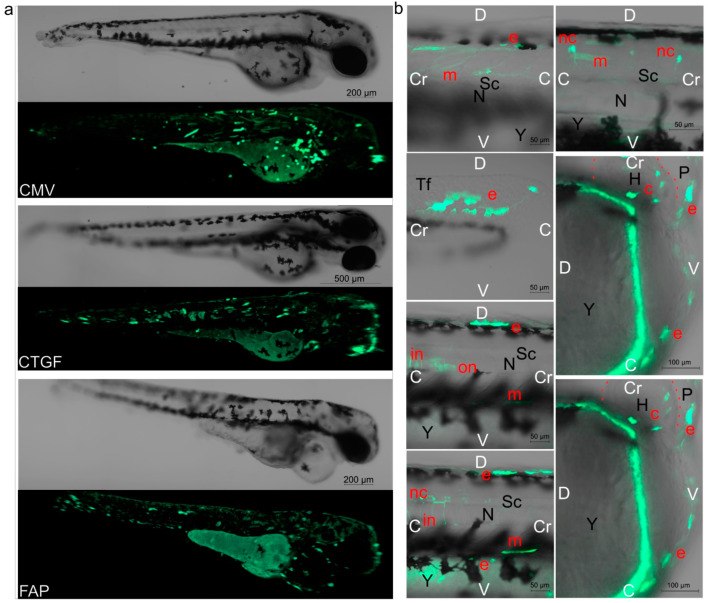
EGFP fluorescence in embryos, 72 h after injection. (**a**) Distribution of EGFP+ fluorescence in embryos injected with 30 attomoles of pGL3(CMV-EGFP), pGL3(CTGF-EGFP), or pGL3(FAP-EGFP). The images shown are obtained by overlaying photographs while scanning along the Z-axis. (**b**) Photographs of individual EGFP+ cells from various body parts of several embryos after injection of 30 amole pGL3(CMV-EGFP). Detailed imaging of labeled embryonic cells using *FAP* and *CTGF* promoters in vectors is not shown. White marks: Cr—cravial pole, C—caudal pole, D—dorsal pole, V—ventral pole. Black marks: N—notochord, P—pericardium, Y—yolk sac, Tf—tail fin, H—heart, Sc—spinal cord. Red marks: e—epithelial cells, m—myocytes, nc—cells of the nervous system, c—cardiomyocytes, on—cells of the outer notochord sheath, in—cells of the inner notochord sheath. Cardiomyocytes and blood cells were identified by their motility during the microscopic analysis. In (**b**), the image of the heart in a state of relaxation and contraction is shown (red dotted contours of the location), muscle cells change their position. Blood cells were evaluated and calculated directly during the analysis.

A bioimaging analysis of EGFP-positive embryonic cells was performed to identify cell populations expressing the marker protein under the control of the analyzed promoters. A mosaic marker expression was observed after the injection of pGL3(CMV-EGFP), pGL3(CTGF-EGFP), or pGL3(FAP-EGFP) vectors into zebrafish fertilized eggs (Figure 2a). Visual morphological analysis was carried out directly in a living organism using non-fixed embryos to identify EGFP-positive embryonic cells. Figure 2b shows the cell populations identified in control embryos after pGL3(CMV-EGFP) injection (detailed imaging of labeled embryonic cells using *FAP* and *CTGF* promoters in vectors is not shown). The injection of the analyzed constructs induced EGFP expression in myocytes (including cardiomyocytes) as well as surface epithelial and outer and inner notochord sheath cells. In the case of CMV and *CTGF* promoters, fluorescence-positive cells were also observed in blood and neural cells.

**Figure 3:**
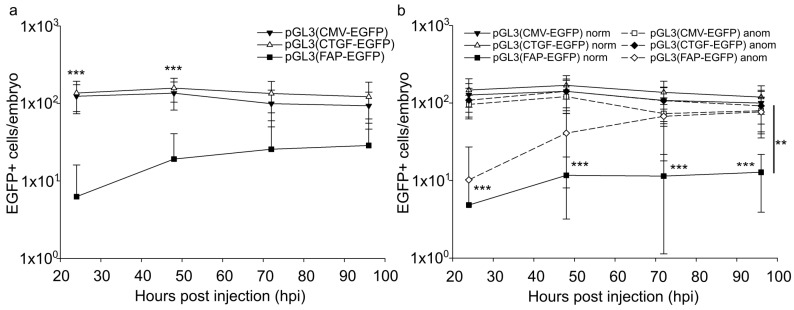
EGFP-positive cells in *Danio rerio* embryos after injection of 30 attomoles of pGL3(CMV-EGFP), pGL3(CTGF-EGFP), or pGL3(FAP-EGFP). The total number of EGFP-positive cells per animal. (**a**) EGFP+ cells in all injected embryos, logarithmic scale. Significant differences in the luciferase activity after the injection of pGL3(CMV-EGFP) and pGL3(CTGF-EGFP) relative to vector pGL3(FAP-EGFP) were observed from 24 to 48 h after injection (*p* < 0.001). (**b**) EGFP+ cells in normal (full lines) and abnormal (dashed lines) injected embryos, logarithmic scale. Abscissa, hours after injection. Significant differences in the number of EGFP-positive cells in normal embryos after the injection of pGL3(CMV-EGFP) and pGL3(CTGF-EGFP) relative to vector pGL3(FAP-EGFP) were observed from 24 to 96 h after injection (*p* < 0.001). The significance of differences in the number of EGFP-positive cells in abnormal embryos was observed 24 h after the injection of pGL3(CMV-EGFP) and pGL3(CTGF-EGFP) relative to vector pGL3(FAP-EGFP) (*p* < 0.001). The significance of differences in the number of EGFP-positive cells in abnormal embryos relative to the one in normal embryos 96 h after the injection of pGL3(FAP-EGFP) were *p* < 0.01. The values were averaged for three independent experiments (*n* = 72 for pGL3(CMV-EGFP), *n* = 78 pGL3(CTGF-EGFP), *n* = 113 for pGL3(FAP-EGFP)). Error bars indicate standard deviation (SD), ** *p* < 0.01, *** *p* < 0.001.

We analyzed the total number of EGFP-expressing embryonic cells, the proportion of such cells with different morphology, and time-related changes in these indices. It should be noted that the number of EGFP-positive cells significantly varied among animal groups, which is likely typical for the model used. After the injection of 30 attomoles of pGL3(CMV-EGFP) or pGL3(CTGF-EGFP), the number of fluorescent cells per animal varied insignificantly in the period from 24 to 96 h after injection (Figure 3a). At the same time, the embryos injected with pGL3(FAP-EGFP) included individuals with the number of EGFP-expressing cells increased 24-fold on average after 3–4 days to approach the levels for other studied promoters (Figure 3b).

Such embryos demonstrated different developmental abnormalities and amounted to 15% of all pGL3(FAP-EGFP)-injected animals or 46% of animals with developmental abnormalities. It should be noted that the proportion of EGFP-positive cells in the rest of the abnormal individuals was similar to that in normal embryos. In the case of pGL3(CMV-EGFP) and pGL3(CTGF-EGFP), the proportion of EGFP-positive cells in abnormal embryos was similar to that in normal ones (Figure 3b).

Thus, the injection of pGL3(CMV-EGFP) or pGL3(CTGF-EGFP) induced a comparable accumulation of EGFP-positive cells in developing zebrafish embryos. In the case of the *FAP* promoter, the number of EGFP-positive cells increased in half of the animals with abnormal development and remained unaltered in normal ones. Apparently, the changed proportion of fluorescent cells in the case of the *FAP* promoter can be due to developmental pathologies at the organism level.

**Figure 4:**
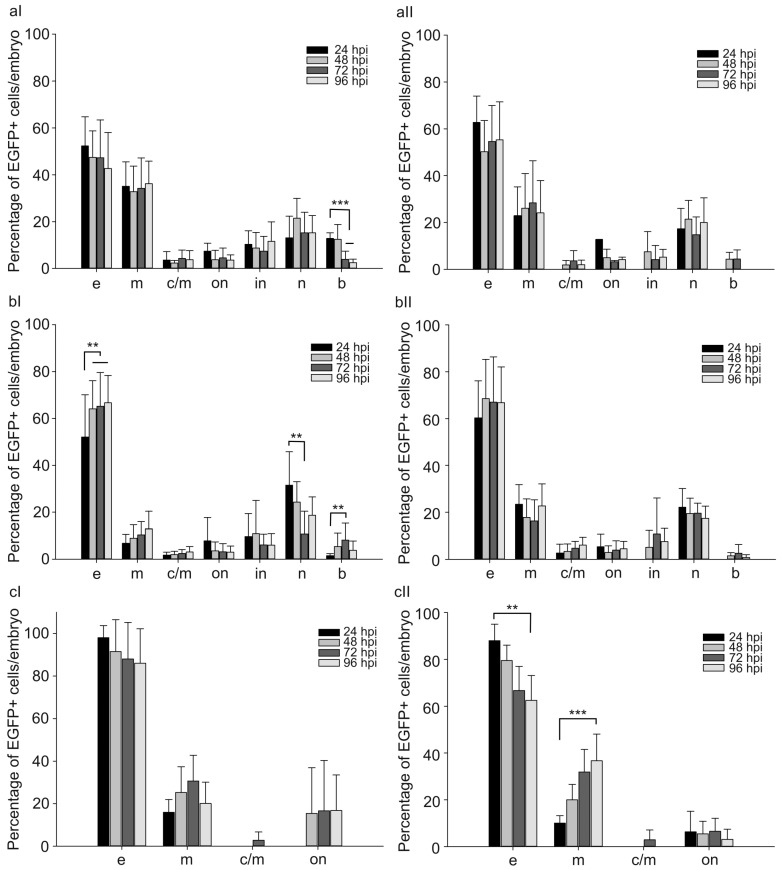
Proportion of EGFP-positive cells of different morphology in normal (**I**) and abnormal (**II**) zebrafish injected with 30 attomoles of (**a**) pGL3(CMV-EGFP), (**b**) pGL3(CTGF-EGFP), and (**c**) pGL3(FAP-EGFP) from 24 to 96 h after injection (hpi). Abscissa, types of EGFP+ cells in injected embryos: e—surface epithelial cells, m—myocytes, c/m—cardiomyocytes, on—outer notochord sheath cells, in—inner notochord sheath cells, n—neural cells, b—blood cells. The values were averaged for three independent experiments (*n* = 62 normal and 10 abnormal animals for pGL3(CMV-EGFP), *n* = 53 normal and 15 abnormal animals for pGL3(CTGF-EGFP), *n* = 85 normal and 28 abnormal animals for pGL3(FAP-EGFP)). Error bars indicate standard deviation (SD), ** *p* < 0.01, *** *p* < 0.001.

Furthermore, the proportions of cells with different morphology were evaluated among normal and abnormal embryos within 4 days after the injection. For CMV and CTGF promoters, the majority of EGFP-positive cells included dermal (50–70%), muscle (20–30%), and nerve cells (10–20%) (Figure 4a,b). Other EGFP-positive cells amounted to at most 10%. In the case of pGL3(CMV-EGFP) or pGL3(CTGF-EGFP) injection, the proportions of different EGFP-positive cells insignificantly varied with time and were similar in both normal and abnormal developing individuals (Figure 4a,b). In the case of the *FAP* promoter, the mean proportions of EGFP-positive dermal and muscle cells amounted to 90 and 10%, respectively, 24 h after the injection in all injected animals (Figure 4c). The marker gene expression was rarely observed in chordal cells.

After the injection of pGL3(FAP-EGFP), the proportions of cell populations did not significantly differ in normal and a fraction of abnormal animals with a reduced number of fluorescent cells within 4 days (Figure 4c). At the same time, a group of abnormal embryos with numerous fluorescent cells demonstrated altered proportions between morphological groups of EGFP-positive cells in the period from 24 to 96 h after injection.

The proportion of fluorescent muscle cells increased from 10 to 35%, while that of dermal cells decreased from 90 to 60%. Hence, a change in the ratio of EGFP-positive populations is observed in this group of embryos during the analysis. Presumably, the effect of the *FAP* promoter is most pronounced in muscle cells.

### 2.3. Lethality and Abnormality Rates in Developing Embryos Injected with Studied Vectors

**Figure 5:**
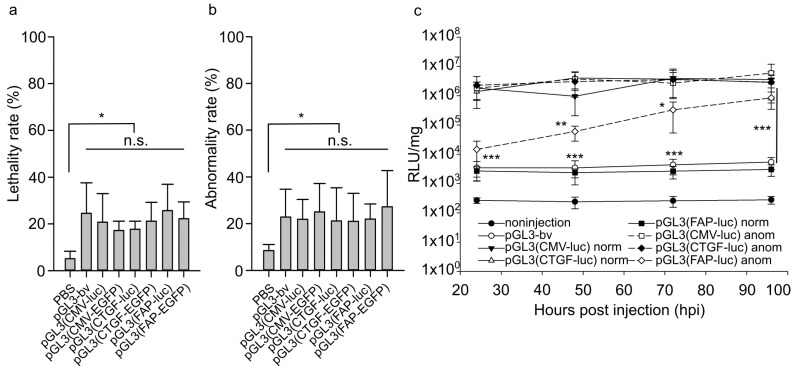
Lethality (**a**) and abnormality (**b**) rates in developing zebrafish embryos 24 h after injection of 30 attomoles of pGL3(CMV-EGFP), pGL3(CTGF-EGFP), pGL3(FAP-EGFP), pGL3(CMV-luc), pGL3(CTGF-luc), pGL3(FAP-luc), pGL3-bv, and buffer solution. Ordinate, the proportion of surviving animals relative to injected ones or proportion of abnormal animals relative to surviving ones 24 h after injection. The values for the uninjected group were taken as the baseline. The data were averaged for three independent experiments. (**c**) Luciferase activity in normal and abnormal zebrafish embryos injected with 30 attomoles of pGL3(CMV-luc), pGL3(CTGF-luc), pGL3(FAP-luc), or pGL3-bv. Abscissa, hours after injection; ordinate, relative luminescence units (RLU) normalized to 1 mg total protein in embryonic lysates, logarithmic scale. The dashed lines indicate the luciferase activity in abnormal individuals. The values were averaged for three independent experiments with three replicates for each point (*n* = 27). Significant differences in the luciferase activity in normal embryos after the injection of pGL3(CMV-luc) and pGL3(CTGF-luc) relative to vector pGL3(FAP-luc) were observed from 24 to 96 h after injection (*p* < 0.001). The significance of differences in the luciferase activity in abnormal embryos was observed 24 h (*p* < 0.01), 48 h (*p* < 0.01), and 72 h (*p* < 0.05) after the injection of pGL3(CMV-luc) and pGL3(CTGF-luc) relative to vector pGL3(FAP-luc). The significance of differences in the luciferase activity in abnormal embryos relative to the one in normal embryos from 48 to 96 h after the injection of pGL3(FAP-luc) was *p* < 0.001. Error bars indicate standard deviation (SD), * *p* < 0.05, ** *p* < 0.01, *** *p* < 0.001; n.s.—not significant for all columns in the specified range.

The effect of promoter sequences on zebrafish development was evaluated using the embryonic survival and abnormality rates after the injection of the vectors (Figure 5a,b). A developing embryo with any body part deformed or absent was considered abnormal. In these experiments, the mean number of dead uninjected embryos 24 h after fertilization was 15%, and abnormal embryos amounted to about 10% of the surviving ones.

The injection of the buffer solution into zebrafish eggs increased by 5% both the number of dead embryos and the rate of defective individuals, which can be attributed to the injection injury (Figure 5a,b). After the injection of pGL3(CMV-EGFP), pGL3(CTGF-EGFP), pGL3(FAP-EGFP), pGL3(CMV-luc), pGL3(CTGF-luc), pGL3(FAP-luc), as well as the control pGL3-bv, the survival rate decreased by ~10–15% relative to animals injected with the buffer solution, while the rate of abnormal development increased by 15–20% in all cases. No significant difference in embryotoxicity between the used genetic constructs was revealed. The data obtained indicate that analyzed promoters do not affect the model organism’s development.

### 2.4. Reporter Protein Accumulation in Abnormally Developing Injected Animals

*Photinus pyrales* luciferase gene was used to evaluate the expression difference between the normal and abnormal zebrafish embryos. Injection of pGL3(CMV-luc) or pGL3(CTGF-luc) yielded similar luciferase activities in normal and abnormal animals (Figure 5c). The vector with the *FAP* promoter also induced no changes in normal embryos relative to the reference vector pGL3-bv. At the same time, a prominent increase in luciferase activity was observed in abnormal animals (Figure 5c), which can indicate the activation of the exogenous *FAP* promoter.

## 3. Discussion

The goal of this study was to analyze the functioning of regulatory elements of the human *FAP* and *CTGF* genes at the organism level. Experiments were conducted on developing zebrafish at late embryonic and early larval stages.

It should be noted that in studies in zebrafish, when using vector systems with regulatory elements of other animal species, the expression of target genes is also found in tissues nonspecific for the promoters used, in addition to the characteristic cellular pattern [34,35,36,37]. This can result from the effect of zebrafish-specific transcription factors on the function of studied expression constructs, and it should be considered when using this model. Nevertheless, this developing zebrafish model can provide new data on the vector function at the organism level and could be employed to compare the efficiency of human *FAP* and *CTGF* promoters in a multi-population system [33,34,35].

The core of the regulatory element of the *CTGF* gene (408 bp fragment, −365/+43 relative to the transcription start site (TSS)) was used in experiments, which is a necessary and sufficient promoter sequence [26,31,32]. Conflicting data are available on the relationship between the *FAP* regulatory element length and efficiency of target gene expression, which may depend on the cell culture used [32,38,39]. Here, the *FAP* promoter length was 2144 bp (−2026/+118 relative to the TSS). The studied regulatory elements in the in vitro system reduced the reporter gene expression relative to the promoter of immediate early genes of *Human cytomegalovirus* [31,32]. The *CTGF* and *FAP* promoters provided 5 to 20 times and ~700 times lower reporter activity relative to the CMV promoter, respectively [32]. The construct with the *CTGF* promoter induced a pronounced relatively steady luciferase activity in the embryo 24 to 96 h after injection into fertilized zebrafish eggs. The observed luciferase activity for the *CTGF* promoter did not significantly differ from that of the CMV promoter in the studied dose range from 0.3 to 30 attomoles.

Similar results were obtained by the quantitation of EGFP-positive cells and analysis of time-related changes in the number of fluorescent cells in embryos injected with constructs with these promoters. Bioimaging analysis revealed no differences in patterns of morphologically different cell populations expressing the marker protein under the control of CMV and *CTGF* promoters. These data indicate that the *CTGF* and CMV promoters within the expression construct provided for similar functional efficiency at the organism level.

The *FAP* promoter demonstrated a different pattern at the organism level compared to *CTGF*. Luciferase activity significantly increased in the lysates of zebrafish embryos from 24 to 96 h after fertilization when vectors with the *FAP* promoter were used. Bioimaging analysis demonstrated that, although the morphology of fluorescent cells was indistinguishable for the constructs with the *FAP* and CMV promoters, a fraction of individuals with abnormal development demonstrated a high number of EGFP-positive cells. At the same time, the proportion between fluorescent cells of different morphology also changed in such embryos. In particular, the proportion of muscle cells increased at a higher number compared to other EGFP-positive cells, which can indicate more prominent changes in the exogenous *FAP* promoter activity in these cells. Likewise, the luciferase activity increased in the lysates of abnormally developing embryos, while it remained steady in normal ones. The data obtained suggest that elevated marker gene expression under the control of the human *FAP* promoter was associated with abnormal embryonic development.

Our study has demonstrated the activation of the human *FAP* promoter in a plasmid vector at the organism level. Notably, the *FAP* promoter hyperactivity was only observed in animals with abnormal development. The activity of the expression constructs containing this promoter element induced no extra pathologies in the model organism as indicated by the embryotoxic analysis. Thus, abnormal tissue formation is the factor modulating *FAP* promoter activity in the vector.

The revealed phenomenon can be related to the *FAP* gene function in vertebrates. The fibroblast activation protein participates in ECM remodeling and stromal structure formation. Elevated FAP expression accompanies the processes that require or follow such changes. These include the regeneration of damaged tissues [40] as well as decidualization in early pregnancy [41] and embryogenesis [42]. Activation of the human native *FAP* gene is also observed in certain pathologies (fibrosis [20], inflammation [21,22], and oncogenesis [23,24,27]) with abnormal stromal reorganization induced by external or internal factors, which can be the case in defective model animals. In this context, the *FAP* promoter activation within the vector agrees with the natural pattern of the gene function, and the data obtained reflect the function of the construct with this element in the atypical stroma.

Thus, the data obtained with the zebrafish model allow us to consider the promoters of the human *FAP* and *CTGF* genes as promising for constructing stromal-oriented vector systems. We believe that these elements can be used in plasmid vectors (as demonstrated in this work) as well as in viral, minimized, etc. ones. However, possible limitations should be considered. The *FAP* and *CTGF* genes are not strictly tissue-specific [25,28,29,30]; hence, marginal expression of the therapeutic agent in untargeted cell populations should be taken into account. In this context, specific carriers for the genetic construct delivery should be used to increase their accumulation in the required tissues or the vectors should be introduced directly into the damaged area.

## 4. Materials and Methods

### 4.1. Genetic Constructs

Two sets of genetic constructs based on the commercial pGL3 vector (Promega, Madison, WI, USA) were used in experiments. The expression cassette included the *Photinus pyrales* luciferase gene or enhanced green fluorescence protein gene under the control of the constitutive promoter of immediate early genes of *Human cytomegalovirus*, promoter of the human fibroblast activation protein gene −2026/+118 relative to the TSSor the core of the regulatory element of the human connective tissue growth factor gene (−365/+43 relative to the TSS). The expression vectors with the luciferase gene as the reporter were constructed previously [32].

The constructs with the *EGFP* gene under the control of *FAP* or *CTGF* promoters were derived from the pGL3(CMV-EGFP) described previously [32].

The pGL3(FAP-EGFP) vector was generated by cloning the *EGFP* gene with the late poly(A) site of *SV40* from pGL3(CMV-EGFP) into the *NcoI* and *SalI* sites of pGL3(FAP-luc). pGL3(CTGF-EGFP) was generated in a similar way using the *NcoI* and *XbaI* sites. The commercial pGL3-basic vector (Promega, Madison, WI, USA) without the regulatory elements was used as a control.

The plasmids were used to transform *E. coli* TG1 cells (from the collection of the Institute of Molecular Genetics of the National Research Centre “Kurchatov Institute”, Moscow, Russia) by electroporation using a MicroPulser (Bio-Rad Laboratories, Hercules, CA, USA). The plasmids were isolated using a Plasmid Miniprep kit (Evrogen, Moscow, Russia). DNA concentrations were determined spectrophotometrically using an extinction coefficient of 0.02 mL/(μg × cm) for double-stranded DNA [43].

### 4.2. Organismic Model

The wild-type AB strain of *Danio rerio* was used. Fish were kept in a flow-through aquarium (Aqua Schwarz, Göttingen, Germany) at 26–28 °C. The light:dark cycle was 14:10 following the international standard. Fish were fed once a day with nauplii *Artemia salina* (Barrom, Barnaul, Russia) or dry food (Sera Vipan, Immenhausen, Germany).

The study was conducted according to the regulations of the European Convention for the Protection of Vertebrate Animals used for Experimental and Other Scientific Purposes (ETS No. 123) and bioethical principles.

### 4.3. Vector DNA Microinjection into Danio rerio Eggs

DNA samples were dissolved in PBS (Biolot, Moscow, Russia) containing 0.05% Phenol red dye (Sigma-Aldrich, St. Louis, MO, USA). DNA concentration ranged from 0.3 to 30 attomoles/nL. DNA samples were microinjected into zebrafish embryos at the first cleavage 20 min after fertilization using an M-152 micromanipulator (Narishige, Tokyo, Japan) and an air-pressure injector PicoPump PV820 (World Precision Instruments, Sarasota, FL, USA) under an inverted microscope Olympus IX2-SLP (Olympus, Tokyo, Japan). The samples (1 nL) were injected within 0.28 s into the yolk under the formed germinal disc at an angle of 45° to the plate to maximize sample delivery into the yolk center. The capillaries used with an outer diameter of 20 µm were pulled from glass capillaries (BF100-50-10, Sutter Instrument, Novato, CA, USA) by a Micropipette puller (Sutter Instrument, Novato, CA, USA). A total of 6750 eggs were injected.

### 4.4. Quantitation of Embryotoxic Effect of Vector DNA Injection into Zebrafish Embryos

The development of *Danio rerio* embryos was monitored under an inverted microscope Olympus IX2-SLP. A total of 24 h after fertilization, their survival rate and the number of abnormal embryos were evaluated. A developing embryo with deformations or absence of the brain segment, complete or partial absence of the caudal section, circulation disorders (pericardial edema and abnormal vascular circulation and structure), sagittal and air bladder defects, abnormal body development (spinal curvature, achordia, and growth retardation), yolk sac defects (edema or developmental abnormalities), or developmental delay was considered abnormal.

In each experiment, 30 uninjected embryos were used as the control. The total number of fertilized eggs was taken as 100%. The proportion of surviving embryos was calculated by taking the number of injected fertilized eggs as 100% in each experiment. The proportion of surviving embryos after injection was calculated by taking the number of injected fertilized eggs as 100%. In the lethality plot, we subtracted the proportion of dead control embryos from these values to take the lethality rate in uninjected animals as the zero level.

The total proportion of abnormal embryos was calculated relative to the number of animals that survived 24 h after injection. In the diagram of abnormal animal proportions, we subtracted the percentage of defective animals in the control group from the values obtained to take the number of defective individuals among uninjected animals as the zero level. The method of embryotoxicity assessment was described in detail elsewhere [44].

### 4.5. Quantitation of Specific Luciferase Activity in Zebrafish Embryos

On average, 100 eggs were injected in each experimental group and about 50 eggs for the control uninjected group. Specific luciferase activity was calculated using the total protein level determined by the modified Bradford method and the luminescence level according to the protocol of the Luciferase Assay System (Promega, Madison, WI, USA) in zebrafish cell lysates [45,46].

Cell lysates were obtained from dechorionated embryos anesthetized with 0.006% aqueous tricaine (Sigma-Aldrich, St. Louis, MO, USA). The embryos were transferred to microcentrifuge tubes (3 embryos per tube; Scientific Specialties, Lodi, CA, USA) removing as much tricaine solution as possible, and 50 μL of lysis buffer (Luciferase Assay System Kit, Promega, Madison, WI, USA) was added with glass powder (G4649, Sigma-Aldrich, St. Louis, MO, USA). The embryos were homogenized using an Eppendorf micropestle (Thermo Fisher Scientific, Waltham, MA, USA) and spun in a microcentrifuge at 13,000 rpm for 15 min. The resulting supernatant was used to assay luciferase activity and total protein in a 96-well plate Costar 3912 or 9017, respectively (Corning, New York, NY, USA), using an Infinite M200 PRO plate spectrophotometer (Tecan, Zürich, Switzerland).

The protein quantitation solution contained 0.03% Coomassie G-250 (LOBA, Fischamend, Austria), 5% ethanol, and 10% phosphoric acid (Chimmed, Moscow, Russia). The calibration curve was plotted using bovine IgG (Reanal, Budapest, Hungary) as the standard.

The substrate solution for the luciferase activity assay included 1 mM D-luciferin of *Photinus pyrales* (Promega, Madison, WI, USA), 25 mM Tris-phosphate, 50 mM 2-mercaptoethanol, 2.5 mM EDTA, 10 mM of MgSO_4_, (Amresco, Radnor, PA, USA), and 4 mM dATP (AppliChem, Darmstadt, Germany), pH 7.8.

### 4.6. Detection of EGFP-Positive Cells in Developing Zebrafish Embryo and Bioimaging

Identification of cell populations expressing EGFP and EGFP-positive cells in counting was performed by visual inspection. On average, 50 eggs were injected in each experimental group and about 20 eggs were selected in the uninjected control. Bioimaging analysis was performed using an upright fluorescence microscope Leica DM 1000 with a fluorescence module Leica GFP ET (Ex 470/40 nm, Em 525/50 nm) and slides (Leica Microsystems, Wetzlar, Germany).

Image acquisition was performed using a confocal laser scanning microscope Zeiss M900 (Carl Zeiss, Oberkochen, Germany). Prior to imaging, the embryos were anesthetized with a 0.006% aqueous solution of tricaine (Sigma-Aldrich, St. Louis, MO, USA) and transferred to a confocal microscopy dish (SPL Life Sciences, Pocheon, South Korea) with a 3% aqueous methylcellulose (Sigma-Aldrich, St. Louis, MO, USA). The images were processed using the ZEN 3.4 software (Carl Zeiss, Oberkochen, Germany). An average of 8 uninjected embryos and 13 animals in each injected group were photographed.

### 4.7. Statistical Analysis

Statistical processing of experimental data was performed using MS Excel 2010 (Microsoft, Redmond, WA, USA) and SigmaPlot 14.0 (Systat Software, Chicago, IL, USA). The significance of the difference between groups was evaluated by one-way ANOVA with Tukey’s correction for multiple comparisons. Differences were considered significant at *p* < 0.05.

## 5. Conclusions

In summary, the efficiencies of genetic constructs under the control of promoters of the human genes *CTGF* and *FAP* were compared at the organism level with that of immediate early genes of human *CMV*. Within the studied vector dose range, the *CTGF* and CMV promoters provided similar efficiency at the organism level. No significant difference in the accumulation of the luciferase activity and the number of EGFP-positive cells as a result of the *CTGF* and CMV promoter functioning were observed throughout the observation period.

The expression vectors with the *FAP* promoter ensured a 100-fold lower luciferase activity and a 10-fold smaller number of EGFP-positive cells in normally developing animals. At the same time, the efficiency of the vectors with the *FAP* promoter significantly increased in embryos with developmental abnormalities. As a result, the luciferase activity and the number of fluorescent cells approached those for vectors with the *CTGF* and CMV promoters 96 h after injection. Thus, embryogenetic abnormalities can modulate the function of exogenous *FAP* promoters.

## Data Availability

The data presented in this study are available on request from the corresponding author.
